# Microglia subtypes show substrate- and time-dependent phagocytosis preferences and phenotype plasticity

**DOI:** 10.3389/fimmu.2022.945485

**Published:** 2022-08-29

**Authors:** Shuailong Li, Isa Wernersbach, Gregory S. Harms, Michael K. E. Schäfer

**Affiliations:** ^1^ Department of Anesthesiology, University Medical Center, Johannes Gutenberg-University Mainz, Mainz, Germany; ^2^ Cell Biology Unit, University Medical Center, Johannes Gutenberg-University Mainz, Mainz, Germany; ^3^ Departments of Biology and Physics, Wilkes University, Wilkes Barre, PA, United States; ^4^ Focus Program Translational Neurosciences (FTN), Johannes Gutenberg-University Mainz, Mainz, Germany; ^5^ Research Center for Immunotherapy (FZI), University Medical Center, Johannes Gutenberg-University Mainz, Mainz, Germany

**Keywords:** brain, inflammation, immune response, microglia, phagocytosis, polarization, plasticity, live imaging

## Abstract

Microglia are phagocytosis-competent CNS cells comprising a spectrum of subtypes with beneficial and/or detrimental functions in acute and chronic neurodegenerative disorders. The heterogeneity of microglia suggests differences in phagocytic activity and phenotype plasticity between microglia subtypes. To study these issues, primary murine glial cultures were cultivated in the presence of serum, different growth factors and cytokines to obtain M0-like, M1-like, and M2-like microglia as confirmed by morphology, M1/M2 gene marker expression, and nitric oxide assay. Single-cell analysis after 3 hours of phagocytosis of *E.coli* particles or IgG-opsonized beads showed equal internalization by M0-like microglia, whereas M1-like microglia preferably internalized *E.coli* particles and M2-like microglia preferably internalized IgG beads, suggesting subtype-specific preferences for different phagocytosis substrates. Time-lapse live-cells imaging over 16 hours revealed further differences between microglia subtypes in phagocytosis preference and internalization dynamics. M0- and, more efficiently, M1-like microglia continuously internalized *E.coli* particles for 16 hours, whereas M2-like microglia discontinued internalization after approximately 8 hours. IgG beads were continuously internalized by M0- and M1-like microglia but strikingly less by M2-like microglia. M2-like microglia initially showed continuous internalization similar to M0-like microglia but again discontinuation of internalization after 8 hours suggesting that the time of substrate exposure differently affect microglia subtypes. After prolonged exposure to *E.coli* particles or IgG beads for 5 days all microglia subtypes showed increased internalization of *E.coli* particles compared to IgG beads, increased nitric oxide release and up-regulation of M1 gene markers, irrespectively of the phagocytosis substrate, suggesting phenotype plasticity. In summary, microglia subtypes show substrate- and time-dependent phagocytosis preferences and phenotype plasticity. The results suggest that prolonged phagocytosis substrate exposure enhances M1-like profiles and M2-M1 repolarization of microglia. Similar processes may also take place in conditions of acute and chronic brain insults when microglia encounter different types of phagocytic substrates.

## Introduction

Microglia are central nervous system (CNS) resident cells that originate from fetal macrophages and play an essential role in innate immune responses and CNS homeostasis, both in the healthy and diseased brain (1, 2). Furthermore, it has become clear that microglia can be efficiently targeted by genetic and pharmacological tools (3). As a result, they are considered suitable targets for modulating CNS diseases (4).

Traditionally, two opposing phenotypes of activated microglia, M1-and M2-like, have been described as pro-and anti-inflammatory subtypes analogously to macrophages (5). However, pathological conditions induce different phenotypes of microglia that are unique and distinct from other macrophage cell types, more diverse than an M1/M2 classification, and more heterogeneous than previously anticipated (2, 6–9). This is reflected by the various roles of microglia in CNS homeostasis, comprising beneficial and detrimental actions in CNS diseases after a bacterial infection or acute injuries such as traumatic brain injury and stroke (5, 10–12).

*In vitro* stimulation of microglia with individual growth factors and cytokines can induce M1-like and M2-like microglia with distinct phenotypes and functional properties (8). This also applies to phagocytosis, the main established functional feature of microglia in development, homeostasis, and pathology (13). For example, M2-like microglia induced by anti-inflammatory cytokines IL-4 and IL-10 show overall higher activity in phagocytosis of microbeads than M1-like microglia generated by the pro-inflammatory cytokine IFN-γ (14). Phagocytosis of zymosan, a yeast cell wall component, is increased in IL-4-induced M2-like microglia compared to bacterial endotoxin lipopolysaccharide LPS-induced M1-like microglia (15). M2-like microglia induced by IL-4, IL-13, and IL-10 were also more efficient than LPS/IFN-γ-induced M1-like microglia in the phagocytosis of myelin (16). Likewise, M2-like microglia induced by stem cell factor (SCF) show increased phagocytosis of FITC-IgG opsonized beads compared to GM-CSF-induced M1-like microglia (17). However, other studies reported that GM-CSF-induced M1-like microglia had significantly higher phagocytic activities for FITC-IgG beads than IL-4-induced M2-like microglia (8). Controversial results were also obtained for the phagocytosis of amyloid-β (Aβ). While microglia pretreated with the M1-like-inducing bacterial endotoxin lipopolysaccharide (LPS) display enhanced phagocytosis (18), pro-inflammatory cytokines known to promote an M1-like phenotype inhibited Aβ phagocytosis (19). More recently, treatment of primary mouse microglia either with LPS or synthetic double-stranded RNA poly(I: C) was shown to differently affect phagocytosis of synaptosomes, *E.coli* particles, or IgG beads (20). Microglia also phagocytose stressed or apoptotic neurons which contributes to brain pathology after ischemic injury (21).The receptor tyrosine kinases *Axl* and *Mertk* control the phagocytic specialization of microglia, for example for apoptotic cells generated during neurogenesis (22). Comparative studies on phagocytosis of neurons by different microglia subtypes are scarce. It was shown that LPS- or Aβ-mediated pro-inflammatory stimulation of BV2 microglia induced neuronal loss and death by phagocytosis of neurons (23), while fractalkine (CX3CL1), which promotes M2 polarization (24), increased the phagocytosis of apoptotic neuron-like SY5Y cells *via* Milk Fat Globule Factor-E8 MFG-E8 (25). Overall, these and other studies using distinct microglia subtypes show differences in phagocytic activity *in vitro* but few studies directly compared different microglia subtypes.

In addition, only a few studies have also shown that phagocytosis of specific substrates is associated with changes in microglia phenotypes. For instance, pathogenic oligomeric Aβ shows a more potent induction of M1-like microglia than the fibrillar form (26). Somewhat controversial data were provided for microglia phenotype plasticity after myelin phagocytosis. Myelin enhanced the M1-like profile and dampened the M2-like profile of primary rat microglia (27) but also induced a switch of M1-like microglia to an M2-like state (28). The latter finding may relate to the up-regulation of the scavenger receptor CD36 in macrophages/microglia after myelin phagocytosis (29). Another study using *E.coli* particles, cell debris or Aβ as phagocytosis substrates showed that *E.coli* particles but not the other substrates encountered by microglia triggered secretion of the pathophysiologically relevant matrix metalloproteinase MMP-9 (1). Furthermore, interaction with apoptotic neurons shifts microglia toward distinct remodeling states (30), which share features with disease-associated microglia (31). Finally, phagocytosis of astrocyte- or neuron-derived exosomes may influence microglia polarization due to the transcellular transfer of miRNAs (32, 33)

Taken together, microglia play various roles in innate immune responses, CNS homeostasis and disease, which likely reflect both their heterogeneity and plasticity. However, while their function extends well beyond removing pathogens, dead cells and cell debris, there is still little knowledge about the relationships between microglia subtypes, different types of phagocytic substrates and phenotype plasticity. To address this, we performed *in vitro* experiments with distinct microglia subtypes subjected to phagocytosis assays for different time periods using two types of phagocytic substrates, *E.coli* particles and IgG-opsonized beads, followed by immunocytochemistry, gene expression analyses, nitric oxide assays, and time-lapse live-cell imaging.

## Methods and materials

### Approval of animal experiments

Newborn mice to obtain primary glia were handled in accordance with the institutional guidelines of the Johannes Gutenberg University Mainz, and Rhineland-Palatine, Germany.

### Primary mixed glial culture and differentiation of microglia subtypes

Mixed glial cultures were prepared from cerebral cortices of 1-5 days-old C57BL/6 mice sacrificed by decapitation. Brains were extracted, the meninges were carefully removed, and the cerebral cortex was dissected under a stereomicroscope. Cells were dissociated using the Neural Tissue Dissociation Kit-P according to the manufacturer`s protocol (#130-092-628; Miltenyi Biotec). 3x10^5^ cells/ml were seeded into T25 cell culture flasks in Dulbecco’s modified Eagle medium (DMEM; Life Technologies, Carlsbad) containing 10% fetal calf serum (FCS, Life Technologies, Carlsbad), 1% penicillin/streptomycin (P/S, 100 U/ml, Life Technologies, USA) and cultured at 37°C in a humidified atmosphere of 5% CO_2_ and 95% air (Heraeus^®^ HERAcell^®^ CO_2_ Incubators, Thermo Fisher Scientific, DE). The cultures were maintained for 14 days *in vitro* (div) and the medium was changed every 3 days. Next, cells were detached using Trypsin/EDTA solution (Sigma, Steinheim) and dissociated in medium (DMEM/10% FCS/1% P/S) and 6-7x10^4^ cells per well were seeded in 24-well plates onto Poly-D-Lysine-coated (0.1% PDL Sigma, Steinheim) glass coverslips (13 mm, SCHOTT, Mainz). For the differentiation of microglia subtypes, cells were incubated in medium (DMEM/10% FCS/1% P/S) supplemented with granulocyte colony-stimulating factor and interferon-gamma (GM-CSF: 20 ng/ml; IFNy: 40 ng/ml, PeproTech GmbH, Hamburg), or macrophage colony-stimulating factor and interleukin-4 (M-CSF, IL-4; 20 ng/ml each, PeproTech GmbH, Hamburg) for 7 div to obtain M1-, or M2-like microglia, respectively. Non-supplemented medium was used to obtain M0-like microglia. Medium was replaced by fresh (non-) supplemented medium at 3 div and 7 div. Different treatment conditions of each experiment were run in parallel on the same 24-well plates.

### Immunocytochemistry and morphological assessment

For immunocytochemistry and morphological assessment of microglia subtypes, cultures were fixed with 4% paraformaldehyde (PFA) for 10 min, blocked (5% normal goat serum, 0.5% BSA, 0.1% Triton X-100 in PBS) for 1 h at room temperature (RT), and incubated with primary antibodies specific to Iba1, MHC-II, and MRC1 (**Supplementary Table 1**) overnight at 4°C. The next day, cells were washed with PBS, incubated with fluorophore-conjugated secondary antibodies (**Supplementary Table 1**) for 1 h at RT, washed, and mounted. Images were taken using a confocal laser scanning microscope (LSM5 Exciter; Carl Zeiss DE) with equal acquisition parameters for different microglia subtypes from five independent cell culture preparations (n=5, biological replicates) and five regions of interest (ROIs, n=5) from each coverslip. Morphological parameters (cell size and circularity) of single cells (20-30 cells per ROI) were analyzed using ImageJ (NIH Image, RRID: SCR_003070) with appropriate threshold settings based on anti-Iba1 immunostaining in a blinded and unbiased fashion and data were expressed as mean values from biological replicates (n=5).

### Nitric oxide assay

The Griess assay was used for colorimetric detection of NO_2_^−^ anions which is proportional to nitric oxide (NO) production (34). 200 μL of cell culture supernatants were mixed with 50 μl of 1% sulfanilic acid (Sigma Cat#S9251). Then, 50 μl of 0.1% N-(1-naphthyl) ethylenediamine dihydrochloride (Sigma-Aldrich; Cat#222488) was added, and the absorbance at 540 nm was detected after 10 min using a microplate reader (Sunrise™, Tecan, Switzerland). The nitrite concentration in each sample was interpolated from a standard curve generated from a series of NaNO2 samples (Sigma, Cat#237213) of known concentration.

### Quantitative PCR

The cell culture medium was removed, the cells were rinsed once with PBS, and the RNeasy and QuantiScript Reverse Transcription Kits (Qiagen) were used to extract RNA and transcribe mRNA into cDNA according to the manufacturer’s instructions. The cDNA was amplified and quantified by real-time detection of SYBR Green (Thermo Scientific) with oligonucleotide primers (**Supplementary Table 2**) purchased from Eurofins using the Light Cycler 480 (Roche). For absolute quantification, a target-specific standard curve was generated and the copy numbers of target genes were normalized to the copy numbers of the reference gene *Ppia* (Cyclophilin A) essentially as described (35, 36).

### Phagocytosis assays

Phagocytosis assays were performed using the Red *E.coli* Phagocytosis Assay Kit (PromoKine, Cat#PK-CA577-K964) or the Phagocytosis Assay Kit (IgG FITC complex, Cayman Chemicals, Cat#500290). Phagocytosis substrates were added at dilutions of 1:50 for Red *E.coli* or 1:100 IgG-FITC beads for 3 h or 5 days, respectively, and processed for immunocytochemistry using anti-Iba1or qPCR as described above. To determine the number of microglia with phagocytosis activity, ImageJ was used to outline Iba1-immunolabelled cells followed by counting the number of cells containing Red *E.coli* particles or IgG-FITC beads above a constant threshold level and the percentage of microglia subtypes containing Red *E.coli* or IgG-FITC beads was calculated. To determine microglia phagocytosis capacity in single cells (25 cells per condition), the relative occupancy of Iba1 immunostaining by the fluorescent phagocytosis substrates was calculated. Images were taken in a blinded and unbiased fashion and data were expressed as mean values ± SEM from independent experiments (biological replicates, n=5).

For time-lapse live imaging of microglial cells, primary mixed glial cultures were detached with Trypsin/EDTA solution and 3x10^5^ cells per well were seeded onto 8-well slides (μ-slide, Ibidi GmbH, Germany). Cells were treated with growth factors and cytokines for 7 div as described above to obtain M0-, M1- and M2-like microglia subtypes. A Leica TSP8 confocal laser scanning microscope (Leica Microsystems, Mannheim, Germany) equipped with an incubator module (20% O_2_, 5% CO_2_, and 75% N2, at 37°C) (Oko Labs, Italy) and a motorized position stage were used for time-lapse live imaging experiments. Since CD68 is not involved in binding bacterial/viral pathogens, innate, inflammatory or humoral immune responses (37), we performed live immunolabelling of microglia with rat anti-mouse CD68-BV421 (dilution: 1:125, clone FA-11, BD biosciences, RRID: AB_2744447). The antibody was applied 30 minutes before the random selection of 5 regions of interest (ROI) per condition, followed by the separate addition of the phagocytosis substrates (Rhodamine-*E.coli*: 1:50. IgG-FITC beads: 1:100). The multi-position confocal images were acquired at an interval of 12 mins over 16 hours using a 20x (0.75 NA) planapochromat objective with differential interference contrast imaging. The automated quantification was based on fluorescent particle tracking of the E. coli-rhodamine or IgG-FITC substrates in single cells labelled with BV421-anti-CD68 using Imaris software (version 9.3.1, BitPlane, Zurich, Switzerland). A total of 1-3 x 10^3^ cells were acquired from 5 ROIs of each condition over the 16-hours live imaging period in each of two independent experiments.

### Statistical analysis

Data analyses were performed with GraphPad Prism^®^ (RRID: SCR_002798). Data outliers were identified and removed using ROUT’s test followed by the Shapiro-Wilk test to determine data distribution as specified in the figure legends. Comparisons between two groups were performed dependent on data distribution by Student’s t-test or Mann-Whitney-U test. Multiple comparisons were performed dependent on data distribution by one-way ANOVA (*post-hoc* correction Holm-Šídák) or Kruskal-Wallis test (*post-hoc* correction Dunnett), if F achieved the necessary level of statistical significance p<0.05. Data are expressed as mean ± SEM. Individual data points represent biological replicates or means from biological replicates as specified in figure legends, p*<0.05, p**<0.01, p***<0.001, p****<0.0001.

## Results

### Microglia subtypes differ in morphology, expression of M1/M2 markers and metabolism

**Figure 1:**
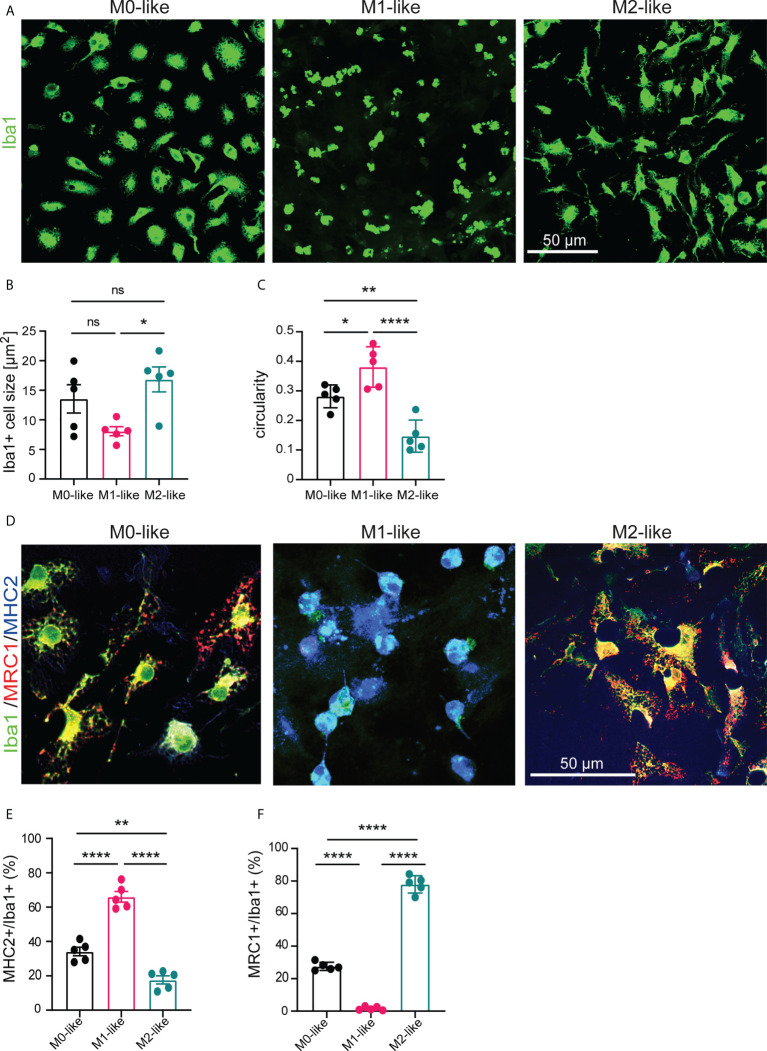
Microglia subtypes differ in morphology and expression of M1/M2 protein markers. **(A)** Confocal images showing anti-Iba1 immunostaining of M0-, M1-, and M2-like microglia subtypes in primary glia cultures at 7 div, cultivated the presence of serum and/or growth factors and cytokines (GM-CSF/IFNγ for M1-like or M-CSF/IL-4 for M2-like) **(B)** Histograms showing differences in mean cell size and **(C)** circularity of microglia subtypes. **(D)** Confocal images showing triple-immunostaining using antibodies specific to the pan-marker Iba1, the M1-marker MHC2, or the M2-marker MRC1. M1- or M2-like microglia show increased expression of MHC2 or MRC1, respectively. **(E, F)** Histograms showing the percentage of Iba1+ microglia expressing MHC2 or MRC1 as determined by cell counts after triple immunostaining using antibodies specific to Iba1, MHC2, or MRC1. Data are expressed as mean ± SEM (n = 5, independent biological replicates are shown) and were tested for significant differences by one-way ANOVA (*post-hoc* correction Holm-Šídák) or Kruskal-Wallis test (*post-hoc* correction Dunnett), *p < 0.05, **p < 0.01, ****p < 0.0001 ns, not significant.

Neural cells were isolated from the cerebral cortex of 3-5 days-old newborn mice, cultivated for 14 days, dissociated and further cultivated in 24 well plates for 7 days in medium (DMEM/10% FCS/1% P/S) containing GM-CSF/IFNy or M-CSF/IL-4 to stimulate proliferation and polarization of microglia into M1-like or M2-like phenotypes, respectively. Heat-inactivated serum was present in all conditions to mimic pathophysiological conditions of BBB breakdown (38, 39) but to prevent complement-enhanced phagocytosis (40). Cultures without the addition of growth factors and cytokines yielded M0-like microglia. First, anti-Iba1 immunostaining to compare microglia morphology between microglia revealed a smaller cell size of M1- than M2-like microglia and a higher circularity of M1-like microglia compared to M0- and M2-like microglia (**Figures 1A–C**). We next assessed the extent of differentiation towards the M1- or M2-like microglia subtype using triple-immunostainings with antibodies specific to the pan-microglia marker Iba1, the M1 marker MHC2, and the M2 marker MRC1 (**Figure 1D**). We found that Iba1^+^/MHC2^+^ cells showed the highest abundance in M1-like microglia (**Figure 1E**, 66.00% ± 3.797%, SEM), an intermediate abundance in M0-like microglia (**Figure 1E**, 34.12% ± 3.797%, SEM), and the lowest abundance in M2-like microglia (**Figure 1E**, 17.65% ± 3.797%, ± SEM). Conversely, the abundance of Iba1^+^/MRC1^+^ cells was highest in M2-like microglia (**Figure 1F**, 78% ± 2.195%, SEM), intermediate in M0-like microglia (**Figure 1F**, 27.52% ± 2.195%, SEM), and almost absent in M1-like microglia (Fig, 1F, 1.651% ± 2.195%, SEM).

**Figure 2:**
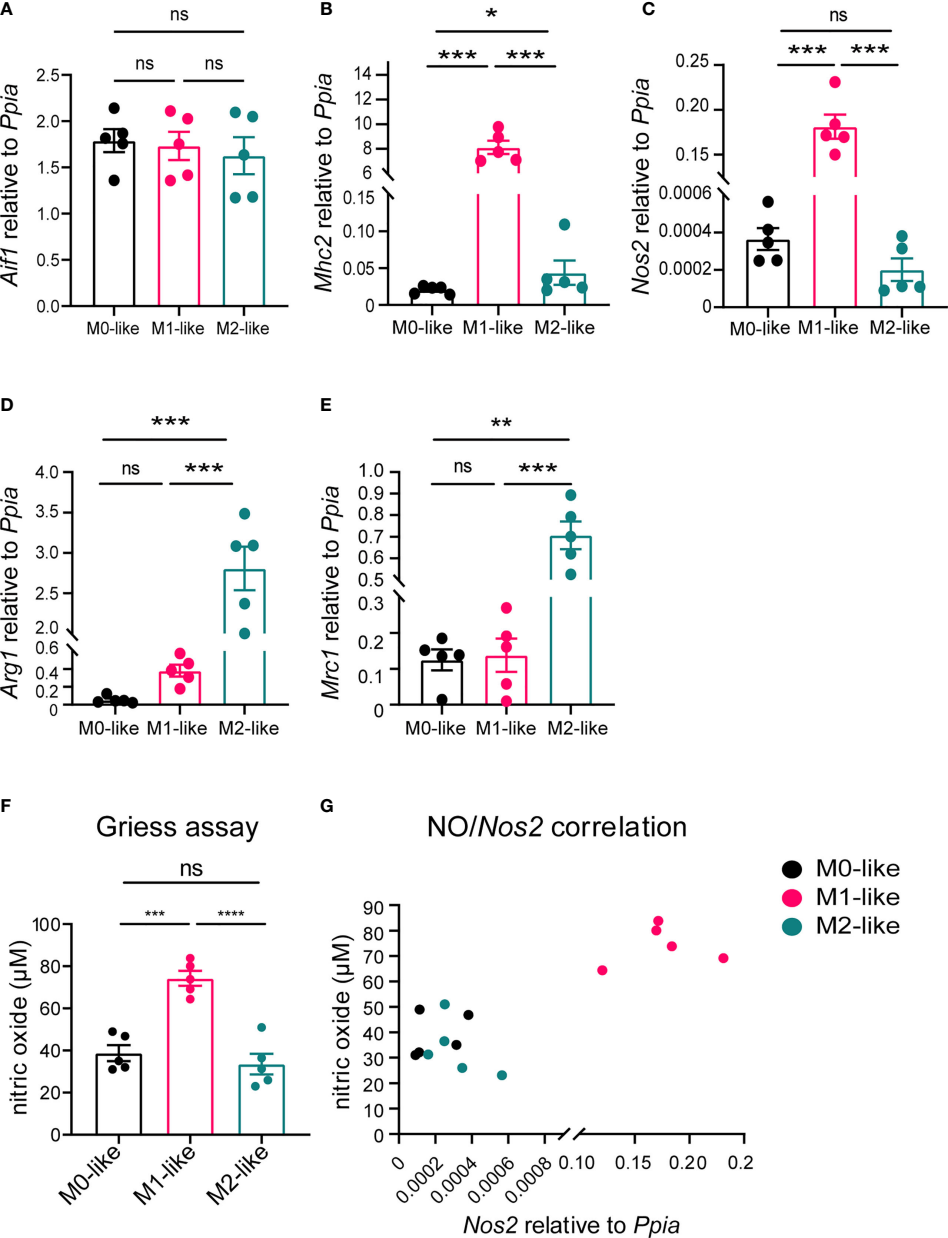
Microglia subtypes differ in M1/M2 gene marker expression and metabolism. **(A–E)** Gene expression analyses of the pan-marker *Aif1*, M1-markers *Mhc2*, *Nos2* and M2 markers *Arg1*, and *Mrc1* demonstrating differential expression by microglia subtypes. *Ppia* was used as a reference gene. **(F)** Alterations in cellular arginine metabolism were detected by colorimetric Griess assay, with M1-like microglia releasing significantly more NO than M0- or M2-like microglia. **(G)** Scatter plot showing positive correlation between NO and *Nos2* expression (non-parametric Spearman correlation, r = 0.8842, p < 0.0001). Values are expressed are mean ± SEM from 5 individual experiments (biological replicates), one-way ANOVA (*post-hoc* correction Holm-Šídák), *p < 0.05, **p < 0.01, ***p < 0.001, ****p < 0.0001. ns, not significant.

To examine whether microglia subtypes showed corresponding gene expression levels, we performed qPCR using primers specific for the established microglia markers *Aif1* (encoding for Iba1), *Mhc2* and *Nos2* (M1 marker) as well as *Arg1* and *Mrc1* (M2 marker). In agreement with previous studies using purified microglia (41, 42), *Nos2* and *Mhc2* were highly expressed in cultures containing M1-like microglia, whereas *Arg1* and *Mrc1* were highly expressed in cultures containing M2-like microglia (**Figures 2A–E**).

To verify that our differentiation protocol induced subtype-specific alterations in cell metabolism, i.e., arginine metabolism, we determined nitrite levels in cell culture supernatants as a measure for the release NO. Cultures containing M1-like microglia released significantly more NO than those containing M0- or M2-like microglia (**Figure 2F**), and the NO release was correlated with the *Nos2* expression (**Figure 2G**). Taken together, these results confirmed the differentiation of M0-, M1-, and M2-like microglia in our primary mixed glia model.

### M1- and M2-like microglia show substrate-specific phagocytosis preference and capacities

**Figure 3:**
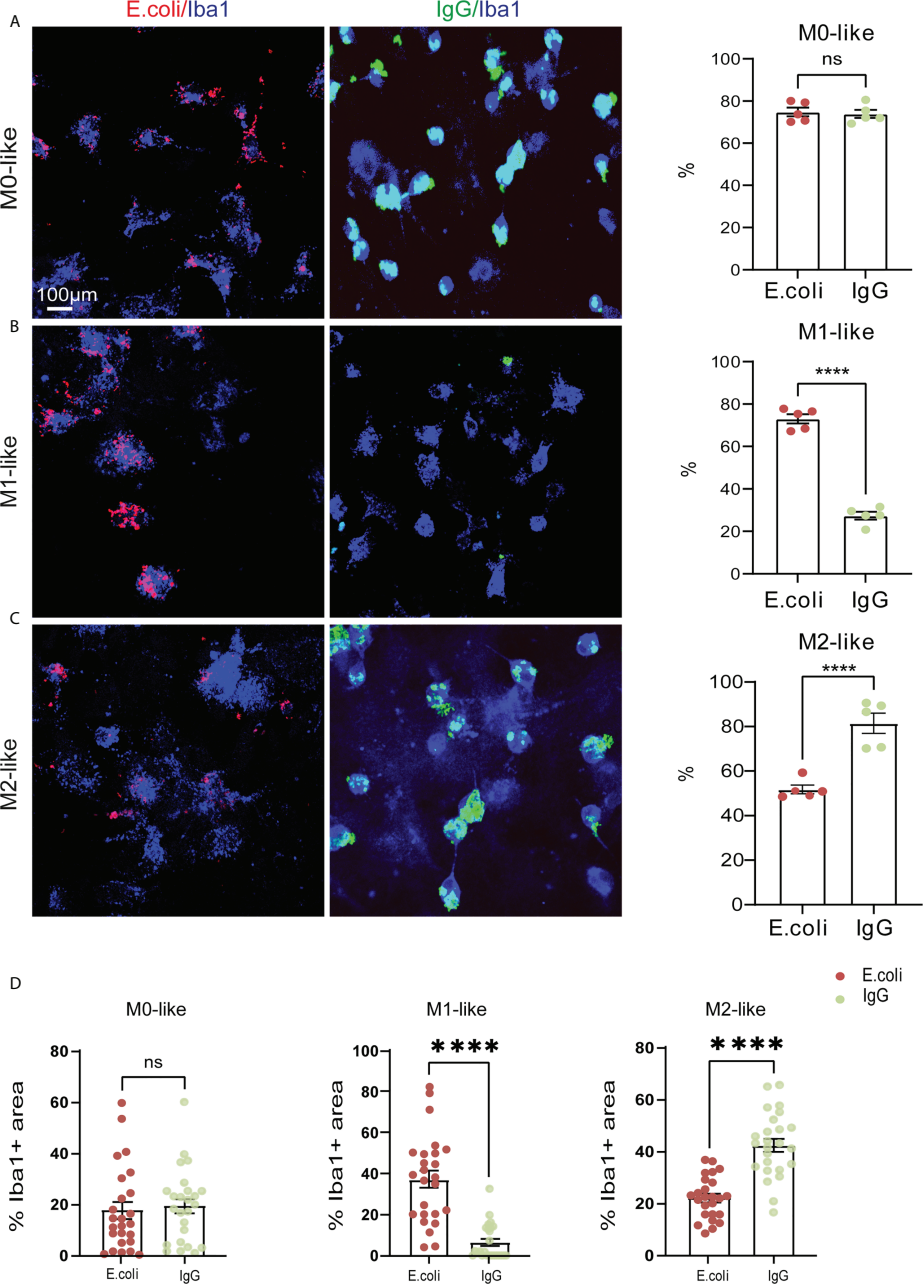
M1- and M2-like microglia show substrate-specific phagocytosis preference and capacities. **(A–C)** Confocal images showing anti-Iba1 immunostaining of M0-, M1- or M2-like microglia subtypes along with rhodamine-E.coli particles (red, E.coli) or IgG-FITC beads (green, IgG) after 3 hours of phagocytosis substrate exposure. Histograms showing percentage of microglia with internalized E.coli particles or IgG beads. M0-like microglia showed no phagocytosis preference, whereas M1- and M2-like microglia showed opposing phagocytosis preferences for E.coli particles or IgG beads, respectively. **(D)** Co-localization analyses showing the relative occupancy of Iba1 immunostained cell areas by E.coli particles or IgG beads. M1- and M2-like microglia displayed opposing phagocytosis capacities for E.coli particles or IgG beads, respectively. Data are expressed as mean ± SEM from 5 independent biological replicates **(A–C)** or 5 cells from each of 5 independent biological replicate **(D)**. Data are means ± SEM. ****p < 0.0001, ns, not significant, Student’s t-test.

We next examined phagocytosis activity and capacity of M0-, M1-, and M2-like microglia in mixed glial cultures. Cells were incubated for 3 h in the presence of two phagocytic substrates, rhodamine-E. coli particles or IgG-FITC beads, and then processed for anti-Iba1 immunocytochemistry and confocal microscopy (**Figures 3A–C**).

Determining the percentage of Iba1^+^ M0-like microglia containing E. coli particles or IgG-beads revealed that approximately 75% of M0-like microglia showed phagocytic activity, regardless of substrate identity (**Figure 3A** *E.coli*: 74.32% ± 2.85%; IgG: 73.86% ± 2.85%, SEM). In contrast, the percentage of M1-like microglia containing *E.coli* particles was higher than the percentage of M1-like microglia containing IgG-beads (**Figure 3B**, *E.coli*: 73.06% ± 2.84%; IgG: 27.37% ± 2.84%, SEM). However, we observed opposing internalization ratios for M2-like microglia. The percentage of M2-like microglia containing IgG-beads was increased compared to the percentage of M2-like microglia containing *E.coli* particles (**Figure 3C**, *E.coli*: 81.49% ± 4.92%; IgG: 51.74% ± 4.96%, SEM).

To assess the phagocytic capacity of the microglia subtypes, we determined the relative occupancy of the Iba1-immunostained cell area by either *E.coli*-rhodamine or IgG-FITC beads in individual microglia. In M0-like microglia, no differences were observed in the cell area occupancy between *E.coli*-rhodamine and IgG-FITC beads, both of which show a cell area occupancy of less than 20% (**Figure 3D**, *E.coli*: 17.68% ± 3.15%; IgG: 19.37% ± 2.97, SEM). In M1-like microglia, the cell area occupancy by *E.coli*-rhodamine was about 35% of the cell area, and the occupancy by IgG-FITC was less than 10% (**Figure 3D**, *E.coli*: 37.12% ± 2.31%; IgG: 6.57% ± 2.07%, SEM). In contrast, M2-like microglia displayed a cell area occupancy by *E.coli*-rhodamine of about 20%, whereas the occupancy by IgG-FITC was more than 40% (**Figure 3D**, *E.coli*: 22.16% ± 0.95%; IgG: 42.53% ± 2.18%, SEM). Together, these results indicated that M1- and M2-like microglia subtypes show substrate-specific phagocytosis activities.

### Microglia subtypes show substrate-specific phagocytosis capacities and dynamics over 16 hours

Our experiments to study differences in phagocytosis by microglia subtypes after fixative treatment and immunocytochemistry at a predetermined time point did not allow examination of microglial phagocytosis continuously over time. Therefore, we performed multicolour time-lapse live imaging for 16 hours. To identify microglia, we added BV421-fluorophore-conjugated anti-CD68 to the cultures 30 min before the imaging experiments started with the addition of *E.coli*-rhodamine particles or IgG-FITC beads. The subsequent analysis was based on fluorescent particle tracking of the *E.coli*-rhodamine or IgG-FITC substrates in single cells labelled with BV421-anti-CD68 and expressed as the mean number of particles per ROI using Imaris software (see methods for details).

**Figure 4:**
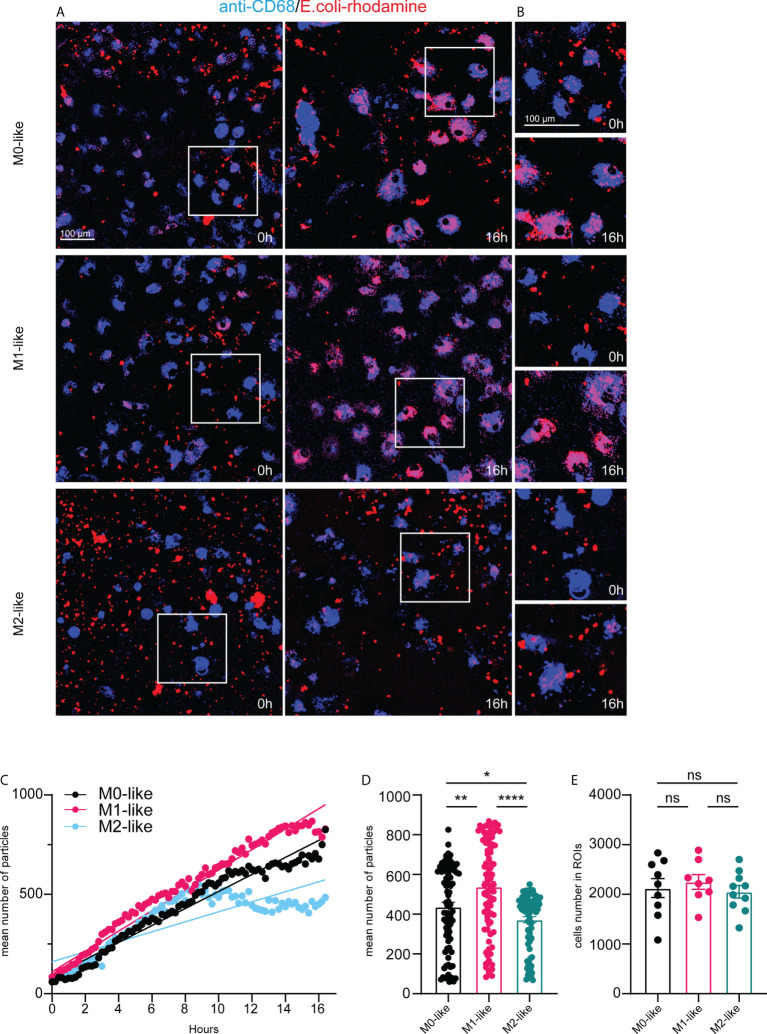
Microglia subtypes show substrate-specific phagocytosis capacities and dynamics for *E.coli* particles over 16 hours **(A)** Single frame images and **(B)** image enlargements from live imaging movies of anti-CD68 live-immunolabelled microglia (blue) after addition of E.coli-rhodamine particles (red) at time-points 0h and 16h, scale bar, 100µm. Note pronounced E.coli particle accumulation in M0- and M1- but not in M2-like microglia at 16h after addition of E.coli particles. **(C)** Time-series plot showing the mean number of E. coli-rhodamine particles internalized (averaged from 8-10 ROIs for each time interval) by microglia subtypes over 16 hours. Simple linear regression calculation indicate different slopes of phagocytic capacities of M0- (black) (r^2^: 0,6902, p < 0,0001), M1- (pink) (r^2^: 0,5364, p < 0,0001), and M2-like (cyan) (r^2^: 0,3691, p < 0,0001), respectively. Note that M2-like microglia discontinued internalization at about 9 hours after addition of E.coli particles. **(D)** Mean number of internalized particles over 16 hours (averaged from 8-10 ROIs for each time interval). **(E)** Number of imaged anti-CD68 immunolabelled microglia encountering E.coli particles over 16 hours. Data are expressed as means ± SEM, one-way ANOVA (*post-hoc* correction Holm-Šídák test, *p < 0.05, **p < 0.01, ****p < 0.0001. ns, not significant.

Images taken shortly after the addition of *E.coli*-rhodamine particles at 0 h and 16 h after their addition indicated high phagocytosis rates of this substrate by M0- and M1-like microglia over time (**Figure 4A**. Higher magnifications demonstrated the internalization of *E.coli* particles into vesicle-like structures and substantial intracellular accumulation of the particles at 16 h (**Figure 4B**). However, M2-like microglia showed less internalized *E.coli* particles than M0- or M1-like microglia (**Figures 4A, B**), suggesting reduced phagocytosis rates over the 16 hours period

*E.coli* particle tracking in CD68+ microglia revealed that M0- and M1-like microglia continuously internalized *E.coli* particles over 16 h. However, internalization by M1-like microglia occurred more steadily (**Figure 4C**). M2-like microglia showed internalization rates similar to M0- and M1-like subtypes until approximately 8 h after adding *E.coli* particles, but then discontinued internalization (**Figure 4C**). These differences were also evident when comparing the mean number of internalized *E.coli* particles (averaged from 3-5 ROIs for each time interval) over 16 h (**Figure 4D**, M0: 445.3 ± 25.80, SEM; M1: 531.9 ± 31.49, SEM; M2: 400.8 ± 18.05, SEM). The mean particle number was significantly higher in M1-like microglia than in M0-like or M2-like microglia, and M0-like microglia showed overall higher internalization than M2-like microglia (**Figure 4D**). These differences were independent of the number of imaged CD68+ cells encountering *E.coli* particles over the 16 h imaging period (**Figure 4E**, M0: 2126 ± 191.7, SEM, M1: 2250 ± 148.9, SEM, M2: 2052 ± 127.8, SEM), which further confirms that the differences between the mean particle numbers per ROI reflect differences at the single cell level.

**Figure 5:**
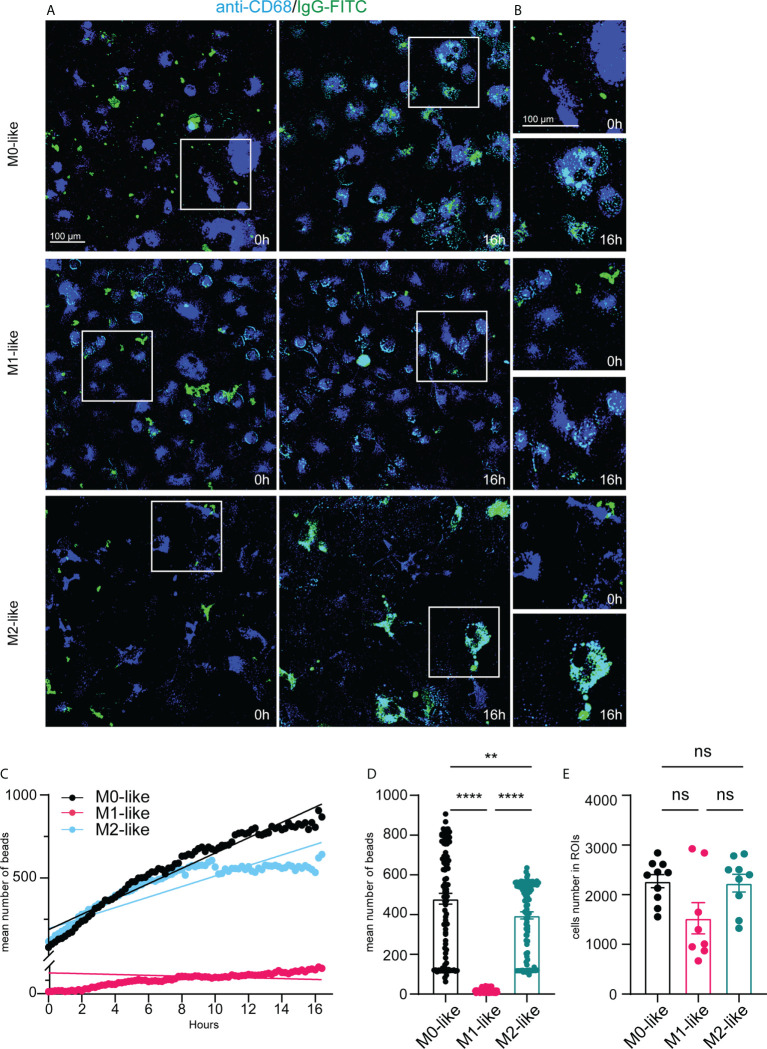
Microglia subtypes show different phagocytosis capacities and dynamics for IgG-FITC beads over 16 hours. **(A)** Single frame images and **(B)** image enlargements from live imaging movies of anti-CD68 live-immunolabelled microglia (blue) after addition of IgG-FITC beads (green) at time-points 0h and 16 h, scale bar, 100µm. Note pronounced IgG bead accumulation in M0- and M2- but not in M1-like microglia at 16h after addition of E.coli particles. **(C)** Time-series plot showing the mean number of IgG beads internalized (averaged from 8-10 ROIs for each time interval) by microglia subtypes over 16 hours. Simple linear regression calculation indicate different slopes of phagocytic capacities of M0- (black) (r^2^: 0,1637, p < 0,0001), M1- (pink) (r^2^: 0,2733, p < 0,0001), and M2-like (cyan) (r^2^: 0,07045, p < 0,0001), respectively. Note that M2-like microglia discontinued internalization at about 9 hours after addition of IgG-FITC beads. **(D)** Mean number of internalized particles over 16 hours (averaged from 8-10 ROIs for each time interval). **(E)** Number of imaged anti-CD68 immunolabelled microglia encountering IgG-FITC beads over 16 hours. Data are expressed as means ± SEM, one-way ANOVA (*post-hoc* correction Holm-Šídák test, ****p < 0.0001. ns, not significant.

Images were taken shortly and 16 h after the addition of IgG-FITC beads to microglia subtypes, suggesting that the phagocytosis of IgG-FITC beads by M0- and M2-like microglia was more efficient than by M1-like microglia (**Figure 5A**).Furthermore, higher magnifications demonstrated the vesicle-like appearance of IgG beads and pronounced accumulation in M0- and M2-like microglia and relatively low accumulation in M1-like microglia (**Figure 5B**).

IgG bead tracking in CD68+ microglia over 16 hours revealed different internalization rates and dynamics by microglia subtypes. M1-like microglia showed continuous internalization but internalized overall, clearly fewer IgG beads than M0- or M2-like microglia (**Figure 5C**). M0-like microglia showed an almost steady increase of IgG bead internalization over 16 h. M2-like microglia initially showed continuous internalization, but discontinuation of internalization occurred approximately 8 hours after adding the phagocytosis substrate (**Figure 5C**). The low phagocytosis activity of M1-like microglia was also reflected by a significantly reduced mean number of internalized beads (averaged from 4-5 ROIs for each 12 min interval) over 16 h, whereas no significant differences were observed between M0-like and M1-like microglia (**Figure 5D**, M0: 443.2 ± 24.81, SEM; M1: 87.27 ± 5.238, SEM, M2: 364.2 ± 17.27, SEM). The number of microglia subtype cells encountering the substrate was statistically not different, albeit the mean number of M1-like cells was lower than in the other conditions (**Figure 5E**, M0: 2273 ± 132.8, SEM, M1: 1532 ± 282.0, SEM, M2: 2231 ± 217.3, SEM).

Altogether, the results indicate that microglia subtypes display substrate-specific phagocytosis over time.

### Microglia subtypes show M1-like features after prolonged phagocytosis substrate exposure

**Figure 6:**
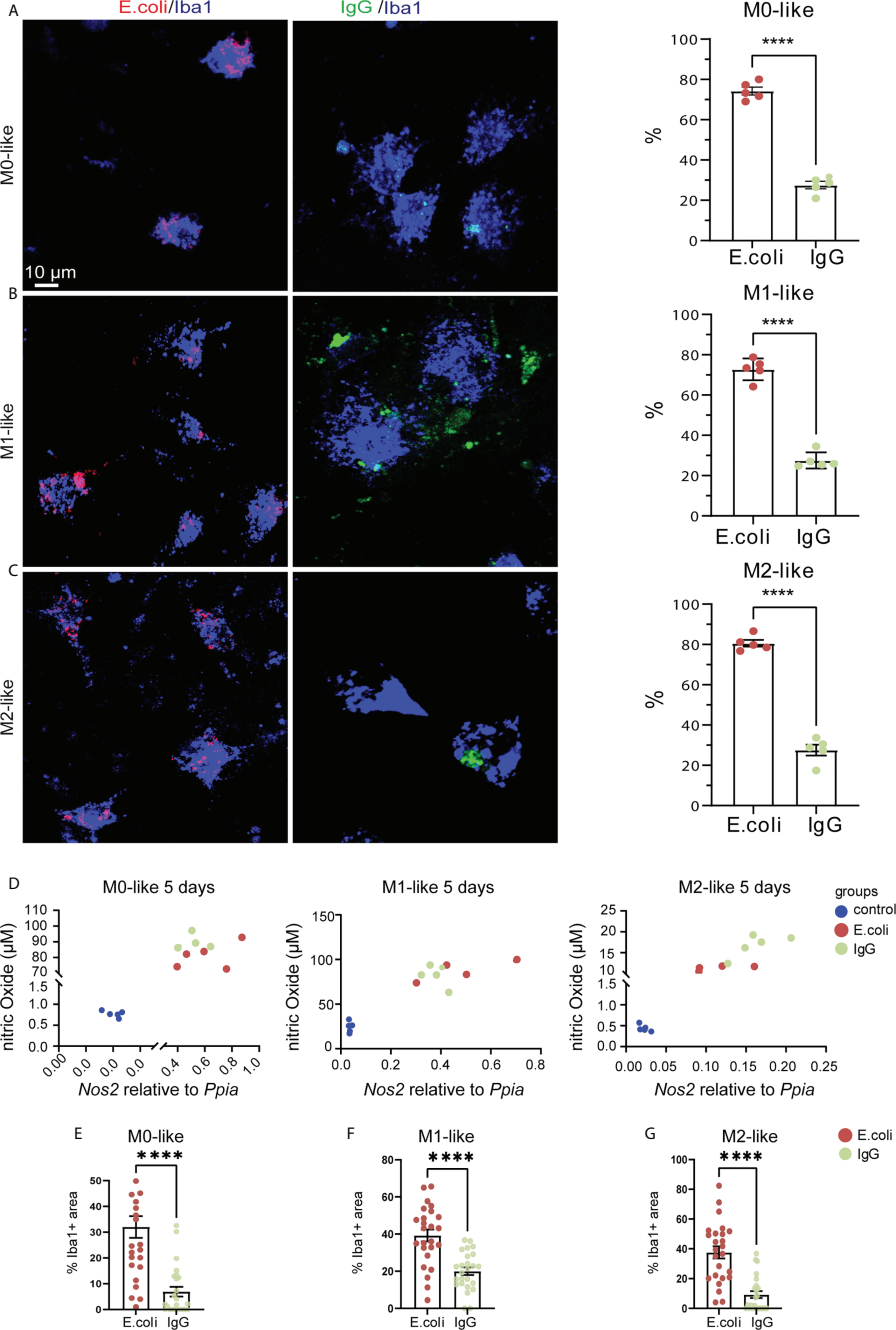
Microglia subtypes show M1-like features after prolonged phagocytosis substrate exposure **(A–C)** Confocal images showing anti-Iba1 immunostaining of M0-, M1- or M2-like microglia subtypes along with rhodamine-E.coli particles (red, E.coli) or IgG-FITC beads (green, IgG) after 5 days of phagocytosis substrate exposure. Histograms showing percentage of microglia with internalized E.coli particles or IgG beads. Microglia subtypes showed phagocytosis preference for E.coli particles rather than IgG beads. **(D)** Scatter plots showing similar correlation (non-parametric Spearman correlation) between NO levels and *Nos2* gene expression for M0- (r = 0.6536, p=0.0099), M1- (r = 0.8214, p = 0.0003), and M2-like (r = 0.8857, p < 0.0001) microglia. Ppia was used as a reference gene. **(E–G)** Co-localization analyses showing the relative occupancy of Iba1 immunostained cell areas by E.coli particles or IgG beads. Microglia subtypes showed phagocytosis preference for E.coli particles rather than IgG beads. Data are expressed as mean ± SEM from 5 independent biological replicates **(A–C)** or 4-6 cells from each of 5 independent biological replicate **(D)**. Data are means ± SEM. ****p < 0.0001, ns, not significant, Student’s t-test.

The phagocytic activity of microglia in neurological conditions may exceed well beyond the time-course examined in our live imaging experiments. To mimic this situation, we subjected microglia to prolonged exposure to phagocytosis substrate for 5 days. Determining the percentage of Iba1+ microglia containing *E.coli* particles after 5 days revealed that approximately 75% of all microglia subtypes showed phagocytosis activity (**Figures 6A–C**, M0: 74.28% ± 4.39%, SEM, M1: 80.6% ± 3.76%, SEM, M2: 72.81% ± 5.4%, SEM). However, the percentage of Iba1+ cells containing IgG beads was significantly lower (**Figures 6A–C**, M0: 27.58% ± 4.16%, SEM, M1: 27.56% ± 6.13%, SEM, M2: 27.55% ± 4.01%, SEM). Analysis of colorimetric NO assays and qPCR results showed that NO release and *Nos2* mRNA expression correlated for all microglia subtypes and that both increased relative to the control microglia subtypes not exposed to phagocytosis substrates (**Figure 6D**). Next, the relative occupancy of the Iba1-immunostained cell area by either *E.coli*-rhodamine or IgG-FITC beads was determined. We found that all microglia subtypes encountering the phagocytosis substrates showed a higher phagocytosis capacity for *E.coli* than for IgG beads (**Figures 6E–G**, *E.coli*: M0: 32.02% ± 4.2%, SEM, M1: 39.22% ± 3.19%, SEM, M2: 37.63% ± 4.06%, SEM; IgG: M0: 6.903% ± 1.9%, SEM, M1: 20.04% ± 2.04%, SEM, M2: 9.307% ± 2.32%, SEM).

**Figure 7:**
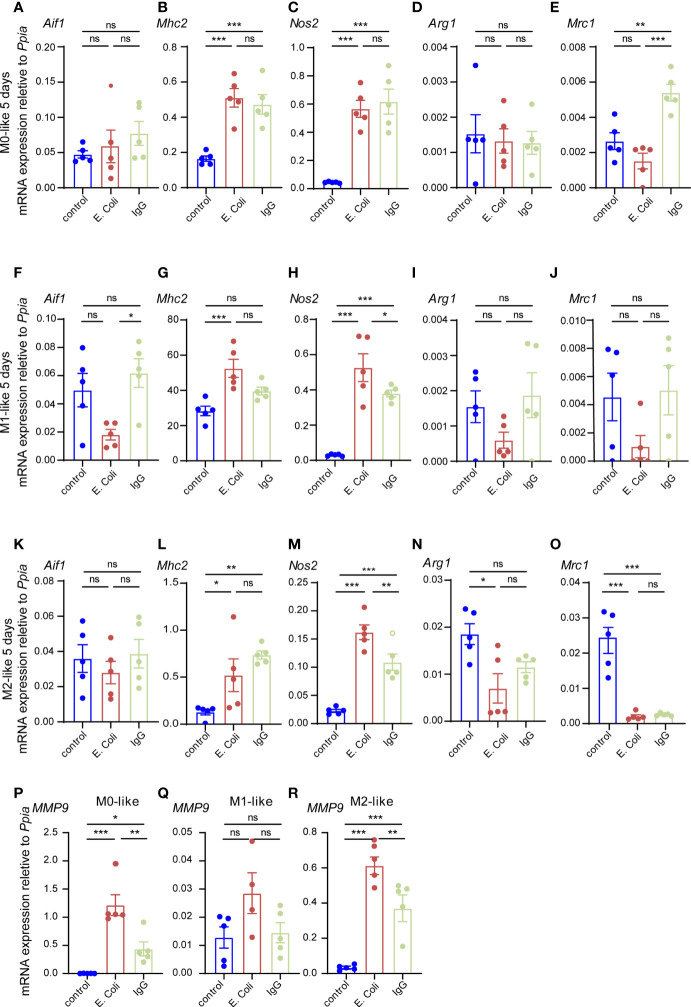
Microglia subtypes show M1-like gene expression after prolonged phagocytosis substrate exposure **(A–R)** Gene expression analyses of microglia subtypes after prolonged phagocytosis substrate exposure (E.coli-rhodamine particles, IgG-FITC beads) for the pan-marker *Aif1*, M1-markers *Mhc2*, *Nos2* and M2 markers *Arg1*, *Mrc1* as well as the glia activation marker *Mmp9*. *Ppia* was used as a reference gene. **(A–E)** In M0-like microglia, conditions with E.coli particles or IgG beads show up-regulation of M1 markers *Mhc2* and *Nos2* as well as up-regulation of M2 marker *Mrc1* as compared to M0-like microglia not exposed to phagocytosis substrate (control). **(F–J)** In M1-like microglia, conditions with E.coli particles show downregulation of microglia pan-marker *Aif1* and up-regulation of M1 markers *Mhc2* and *Nos2* but down-regulation of M2 markers *Arg1* and *Mrc1* and conditions with IgG show up-regulation of *Nos2* as compared to M1-like microglia not exposed to phagocytosis substrate (control). **(K–O)** In M2-like microglia, conditions with E.coli particles or IgG beads show up-regulation of M1 markers *Mhc2* and *Nos2* as compared to M2-like microglia not exposed to phagocytosis substrate (control). Down-regulation of M2 marker *Arg1* was observed in conditions with E.coli particles as well as down-regulation of *Mrc1* in conditions with E.coli particles or IgG beads as compared to M2-like microglia not exposed to phagocytosis substrate (control). **(P–R)** The glia activation marker *Mmp9* was up-regulated in M0- and M2-like microglia both in conditions with E.coli particles of IgG beads as compared to microglia subtypes not exposed to phagocytosis substrate (control). Multiple comparisons were performed dependent on data distribution by one-way Anova (*post-hoc* correction Holm-Šídák) or Kruskal-Wallis test (*post-hoc* correction Dunnett), if F achieved the necessary level of statistical significance p < 0.05. Data points are shown for biological replicates and expressed as mean ± SEM, p* ≤ 0.05, p** ≤ 0.01, p*** ≤ 0.001.

Since this result was in stark contrast to the substrate preferences of microglia subtypes observed in our experiments after 3 h of substrate exposure, we tested whether changes in microglial subtype identity might have contributed to this result. We determined gene expression levels of microglia subtype markers by qPCR using primers specific for *Aif* (*Iba1*), *Mhc2*, *Nos2*, *Arg1* and *Mrc1* (**Figure 7**). In M0-like microglia cultures, the M1-like markers *Mhc2* and *Nos2* were both strongly up-regulated after 5 days of exposure both to *E.coli* particles or IgG beads as compared to M0-like cells cultured without phagocytic substrate (**Figures 7B, C**), while the pan-microglia marker Aif1 was not significantly different between the three conditions (**Figure 7A**). Determination of M2-like marker expression revealed up-regulation of *Mrc1* after IgG bead exposure and *Arg1* expression did not alter in response to substrate exposure (**Figures 7D, E**). In M1-like microglia, we found decreased *Aif1* expression but increased expression of M1-like markers *Mhc2* and *Nos2* after *E.coli* particle exposure as well as increased *Nos2* expression after IgG bead exposure (**Figures 7F–H**). No significant differences were found for *Arg1* or *Mrc1* expression (**Figures 7I, J**). In M2-like microglia, both *E.coli* particles and IgG beads caused up-regulation of M1-like markers *Mhc2* and *Nos2* (**Figures 7L, M**). M2-marker *Arg1* was down-regulated after *E.coli* exposure and *Mrc1* was downregulated both after *E.coli* particle and IgG bead exposure as compared to M2-like cells cultured without phagocytosis substrates (**Figures 7N, O**).

Thus, microglia subtypes subjected to prolonged exposure to phagocytosis substrate for 5 days displayed changes in M1/M2 marker gene expression indicating a shift towards M1-like phenotypes. Recently, it was shown that phagocytosis of *E.coli* particles by M0-like microglia leads to delayed release of the matrix metalloproteinase MMP-9 (43) suggesting that *Mmp9* gene expression may serve as a marker for microglia plasticity in response to phagocytosis substrate exposure. Indeed, *Mmp9* expression was up-regulated in M0- and M2-like microglia after 5 days exposure to *E.coli* particles or IgG beads as compared to control cultures without phagocytosis substrate. However, *Mmp9* expression did not increased significantly in M1-like microglia (**Figures 7P–R**) suggesting that *Mmp9* gene expression was stronger induced in microglia undergoing shifts towards a M1-like phenotype.

## Discussion

This study examined phagocytosis efficiencies of M0-, M1-, and M2-like microglia for *E.coli* particles and IgG-opsonized beads over different time periods *in vitro*. These substrates were chosen due to their pathological relevance, their common use in phagocytosis research and better detection and quantification properties compared to soluble fluorophore-conjugated molecules. Cell cultivation and phagocytosis assays were carried out in the presence of serum to mimic pathophysiological conditions of BBB breakdown such as meningitis, trauma or stroke (38, 39). We found that microglia subtypes differ in phagocytosis efficiencies for the two types of substrates in a time-dependent manner and long-term substrate exposure enhanced or induced M1-like profiles of M0-, M1-, and M2-like microglia, respectively. Our results suggest that phagocytosis substrates can trigger phenotype plasticity of microglia including M2 to M1 repolarization which may also take place in neurological conditions when microglia encounter different types of phagocytic substrates.

In general, our results showing substrate-specific phagocytosis efficiencies of distinct microglia subtypes are consistent with previous *in vitro* studies examining phagocytosis of diverse substrates such as zymosan, IgG-opsonized beads or Aβ by M1- and M2-like microglia (44–48). The prevailing view is that M2-like microglia show higher phagocytic activity than M1-like microglia. However, there are also conflicting observations on the phagocytic preference and efficiency of different microglia subtypes suggesting a context dependency (8, 14, 19, 20, 49, 50).

In the present study, when microglia were exposed to *E.coli* particles or IgG beads for 3 hours, we found that a higher proportion of M1-like than M2-like microglia phagocytose *E.coli* particles. This result is in agreement with findings that M1-like microglia, showing augmented release of NO and expression of pro-inflammatory cytokines, more efficiently phagocytose pathogenic bacteria than unstimulated microglia, but M2-like microglia were not examined (51, 52). Studies comparing *in vitro* phagocytosis dynamics between different microglia subtypes over time are scarce (53). Therefore, we conducted live imaging experiments over 16 hours in anticipation of gaining new insights. Similar to the 3 h exposure time, M1-like microglia internalized more *E.coli* particles over 16 h than M2-like microglia, and *vice versa*, M2-like microglia internalized more IgG beads than M1-like microglia. The strongest differences in substrate preference were found for M1-like microglia, which clearly preferred *E.coli* particles over IgG beads. This result supports and extends the aforementioned findings that M1-like microglia show high efficiency in the phagocytosis of pathogenic bacteria, which holds potential for novel therapeutic approaches e.g. in bacterial meningoencephalitis and sepsis (51, 52, 54, 55). In this context, factors have been identified, i.e. palmitoylethanolamide and activin A, to enhance phagocytosis of *E.coli* by M1-like microglia while preventing excessive and potentially harmful release of NO and pro-inflammatory cytokines (56–58). Interestingly, similar mechanisms may underlie therapeutic benefit after treatment of Alzheimer`s Disease model mice with the non-pyrogenic LPS-derivative monophosphoryl lipid A (59), which promotes phagocytosis of Aβ after 3 h of substrate exposure by pretreated microglia *in vitro* (18). To expand existing research, live cell imaging of microglia over a longer observation time might be useful to characterize this and other phagocytosis-enhancing drugs in terms of optimal stimulation protocols to achieve sufficient phagocytosis activity.

We further found that M2-like microglia internalized IgG beads more efficiently than M1-like microglia. These results are in line with previous studies showing efficient phagocytosis of substrates from other sources than *E.coli* by M2-like microglia (14–16). Likewise, M2-like microglia induced by SCF show increased phagocytosis of FITC-IgG opsonized beads as compared to GM-CSF-induced M1-like microglia (17, 48). However, we found time-dependent changes in substrate internalization of M2-like microglia, almost discontinuing both *E.coli* particle and IgG bead phagocytosis approximately 8 h after addition of the phagocytosis substrates. We have not addressed the question of whether stalled phagocytosis activity by M2-like microglia is a transient or permanent effect beyond the 16 h live imaging period. Interestingly, biphasic phagocytosis activities have been reported for bone marrow-derived macrophages with peaks at 4 h and 24 h and an intervening period of no internalization (60), but the underlying mechanisms are elusive. Rate-limiting factors reported for phagocytosis comprise scavenger receptors, Fcγ and/or complement receptors, the myosin/actin network, second messengers such as phosphoinoside, but also physical and metabolic constraints might play a role (61–64). Further studies using genetic, pharmacological and single-cell transcriptomics approaches are required to modulate key factors of phagocytosis and elucidate the molecular mechanisms underlying this and other observations of our study.

We found that after prolonged substrate exposure for 5 days all microglia subtypes showed a higher preference for *E.coli* particles than IgG beads. Notably, prolonged exposure resulted in increased expression of the M1 markers *Mhc2* and *Nos2* irrespectively of the substrate. Gene expression changes after prolonged IgG bead exposure were similar to those after prolonged *E.coli* particle exposure and in support of a shift towards M1-like phenotypes. These results suggest that phagocytosis associated with the cellular environment can be considered a key factor in the phenotype transformation of microglia. Indeed, it has become clear that phagocytosis is not an isolated cellular response and may represent a source of cellular heterogeneity and plasticity in different tissues (65). Compelling evidence was provided that this is also true for microglia in the CNS. Regional changes in epigenetic regulation of microglia transcriptomes have been connected to the basal phagocytic activity of microglia (66) and phagocytosis-induced transcriptional changes were demonstrated to support the long-term maintenance of hippocampal neurogenesis in mice (67). However, in mouse models of acute or chronic neurodegeneration, phagocytosis of apoptotic cells caused a microglia phenotype shift from a homeostatic to a neurodegenerative phenotype (68). Our results suggest that shifts towards pro-inflammatory M1-like phenotypes occur irrespectively of the pre-established microglia subtype thereby providing another piece of evidence for high microglia plasticity in response to environmental factors. We did not explore whether the presence or phagocytosis of *E.coli* particles or IgG beads were decisive for this phenotype shift but we favor the possibility that both phagocytosis, the environmental presence of substrate as well as the duration of substrate exposure is critical. Indeed, experimental evidence from macrophages shows time-dependent phenotypic switches in response to LPS (69). Dynamic changes in pro-inflammatory cytokine gene expression were also observed in M1-like microglia after myelin phagocytosis (70) and chronic myelin phagocytosis induces a disease-associated transcriptional state in microglia (71). Another non-mutually exclusive possibility is that increased pro-inflammatory cytokine expression mediates feedback loops that enhance or drive a phenotype shift towards M1-like profiles (72–75).

The same could apply to the metalloproteinase MMP-9, which is expressed by LPS-activated microglia in primary neuron-glia cultures (76) and secreted in a delayed manner by primary microglia in response to phagocytosis of *E.coli* particles (43). In support of a role of MMP-9 in microglia responses after phagocytosis, we found robust up-regulation of *Mmp9* expression after long-term substrate exposure for 5 div. Interestingly, *Mmp9* up-regulation was found in cultures showing shifts towards the M1-like phenotype whereas no up-regulation was observed in cultures of pre-differentiated M1-like cells. This finding suggests that MMP-9 may serve as a marker for microglia plasticity including M2 to M1 repolarization, consistent with observations on the positive regulation of MMP-9 expression by M1-like phenotype inducers IL-1, TNFα, and LPS and negative regulation by the M2-like phenotype inducers IL-4 and IL-10 (77). In addition, paracrine/autocrine loops involving MMP-9 have been suggested to amplify microglia activation, whereas deletion of MMP-9 maintained microglia in a resting phenotype in an animal model of spinal cord injury (78). These findings suggest a broader functional spectrum of MMP-9 and other matrix metalloproteinases (MMPs) released by microglia beyond established physiological roles in synaptic plasticity and extracellular matrix modeling (79) or pathological roles in neuroinflammation or gliomas, for example (77, 80, 81).

Our results further support the hypothesis that M1/M2 microglia can shift between functional phenotypes depending upon environmental signals, here *E.coli* particles or IgG beads. Similar processes may also take place in conditions of acute and chronic brain insults when microglia encounter different types of phagocytic substrates. Indeed, M2-like to M1-like shifts in microglia populations were also observed in models of ischemic stroke (82, 83), spinal cord injury (28), and traumatic brain injury (84, 85). Furthermore, phagocytosis by microglia can play an important role in chronic neurodegeneration as well as neurodevelopmental and neuropsychiatric disorders (86–88). Reprogramming patient-derived cells to microglia-like cells and testing for their phenotype plasticity and phagocytosis function may help to gain insights into pathological mechanisms. For example, schizophrenia patient-derived microglia-like cells show higher rates of synaptic phagocytosis and elimination and targeting microglia by the immunomodulatory drug minocycline reduced abnormal synapse elimination by phagocytosis (89). Interestingly, minocycline was proposed to act *via* inhibition of MMPs in the autism spectrum disorder fragile X syndrome (90) suggesting that better understanding the role of MMPs for microglia activation, phenotype plasticity, and phagocytic function may provide novel immunomodulatory treatment options.

Some limitations of this study should be considered. Our *in vitro* approach does not reproduce the brain environment, and many factors influencing microglial morphology, polarization and function are absent. To partially compensate for these limitations, we used primary glia cultures containing a substantial number of astrocytes in combination with microglia-specific immunolabelling as well as microglia-specific qPCR assays. The presence of astrocytes under the different experimental conditions likely influenced microglia responses as compared to pure microglia cultures since astrocytes modulate microglia polarization, activation and function. Conversely, activated microglia can trigger changes in the inflammatory profile of astrocytes both *in vitro* and *in vivo* (91, 92). As LPS-activated microglia can induce a neurotoxic A1 astrocyte phenotype (92), prolonged exposure of the primary glial cultures to *E.coli* particles affects microglia but also astrocytes. Therefore, further studies are required to examine possible alterations in astrocytes and their influence on microglia under the experimental conditions of our study. Another limitation in this study is the use of two different phagocytic substrates and fluorophore conjugates, which likely undergo different lysosomal processing and fluorescent decay after phagosomal acidification. This may particularly play a role for the long-term experiments and the results of the phagocytic uptake should be interpreted with caution. Importantly, regardless of this limitation, data on gene expression and nitric oxide levels demonstrate microglia plasticity and phenotype shifts after long-term substrate exposure. Finally, non-defined serum proteins in the, however heat-inactivated, culture media can trigger microglia activation as well as phagocytosis by microglia (38, 39, 93, 94). As indicated by previous findings, serum-derived IgG likely influenced phagocytosis by microglia in the present studies. It has been also shown that the opsonization of *E.coli* with human serum or murine IgG increases the phagocytic ability of macrophages to clear *E.coli* (60). However, given that the presence of serum mimics neuropathological conditions involving BBB damage in our *in vitro* model, the findings of this study may more closely resemble pathological *in vivo* conditions.

## Data availability statement

The raw data supporting the conclusions of this article will be made available by the authors, without undue reservation.

## Ethics statement

Ethical review and approval were not required for the animal study because we used newborn mice as a source for primary neurons which does not require an ethical review. Newborn mice to obtain primary glia were handled in accordance with the institutional guidelines of the Johannes Gutenberg University Mainz, and Rhineland-Palatine, Germany.

## Author contributions

MS conceptualized and designed the study. SL performed the experiments, data collection, and analysis. IW assisted in experiments and data analysis. GH conducted microscopy methodology and advice on data analysis and interpretation. All authors contributed to the manuscript writing and approved the submitted version.

## Funding

SL is financially supported by the China Scholarship Council.

## Acknowledgments

Data shown in this manuscript are part of the doctoral thesis of SL presented to the Johannes Gutenberg-University Mainz. We gratefully acknowledge the excellent technical assistance of Dana Pieter and Tobias Hirnet.

## Conflict of interest

The authors declare that the research was conducted in the absence of any commercial or financial relationships that could be construed as a potential conflict of interest.

## Publisher’s note

All claims expressed in this article are solely those of the authors and do not necessarily represent those of their affiliated organizations, or those of the publisher, the editors and the reviewers. Any product that may be evaluated in this article, or claim that may be made by its manufacturer, is not guaranteed or endorsed by the publisher.
