# Supplementary material for: Microglia subtypes show substrate- and time-dependent phagocytosis preferences and phenotype plasticity
Source: Front Immunol. 2022 Aug 29;13:945485. doi: 10.3389/fimmu.2022.945485 (PMC9465456; doi:10.3389/fimmu.2022.945485)
Supplement: Supplementary file 1 [file DataSheet_1.docx]

Supplemental Table 1：Primary and secondary antibodies

| **Antibody** | **Host species** | **Dilution** | **Manufacturer (Catalogue#)/RRID** |
| --- | --- | --- | --- |
| Iba1 | guinea pig | 1:500 | Synaptic Systems (#234004)  RRID：AB_2493179 |
| CD68-BV421 rat anti-mouse | rat | 1:125 | BD (#566389)  RRID：AB_2744447 |
| MRC1 | rabbit | 1:200 | MyBioSource (#MBS9129443)  RRID：AB_2916125 |
| MHC-II | rat | 1:500 | Miltenyi (#130-102-186)  RRID:AB_2660056 |
| Alexa Fluor 488 goat anti-guinea pig | goat | 1:500 | Invitrogen/Life Technologies (#A-11073)  RRID:AB_2534117 |
| Alexa Fluor 568 goat anti-rabbit | goat | 1:500 | Invitrogen/Life Technologies (#A-11011)  RRID: AB_143157 |
| Alexa Fluor 633 goat anti-rat | goat | 1:500 | Invitrogen/Life Technologies (#A-21094)  RRID: AB_2535749 |
| Alexa Fluor 633 goat anti-guinea pig | goat | 1:500 | Invitrogen/Life Technologies (#A-21105)  RRID: AB_ 2535757 |

Supplemental Table 2：Oligonucleotide primers

| **PCR assay primer** | **Oligonucleotide sequences 5’-3’** | **Amplicon size (bp)** | **Gene bank number** |
| --- | --- | --- | --- |
| *Arg1* | fw- CTCCAAGCCAAAGTCCTTAGAG  rev- AGGAGCTGTCATTAGGGACATC | 185 | NM_007482 |
| *Aif1/Iba1* | fw- ATCAACAAGCAATTCCTCGAT GA  rev- CAGCATTCGCTTCAAGGA CAT A | 144 | NM_019467 |
| *Mhc2* | fw- CCGCCTAGACAAGCTGACC  rev- ACAGGTTTGGCAGATTTCGGA | 84 | NM_001042605 |
| *Mrc1* | fw- GGCTGATTACGAGCAGTGGA  rev- ATGCCAGGGTCACCTTTCAG | 184 | NM_008625.2 |
| *Nos2* | fw- TGTGTCAGCCCTCAGAGTAC  rev- CACTGACACTYCGCACAA | 312 | NM_010927 |
| *Ppia* | fw- GCGTCTSCTTCGAGCTGTT  rev- RAAGTCACCCTGGCA | 97 | NM_008907 |
| *Mmp9* | fw- AAGTCTCAGAAGGTGGAT  rev- AATAGGCTTTGTCTTGGTA | 106 | NM_013599 |
